# Automated Techniques for the Interpretation of Fetal Abnormalities: A Review

**DOI:** 10.1155/2018/6452050

**Published:** 2018-06-10

**Authors:** Vidhi Rawat, Alok Jain, Vibhakar Shrimali

**Affiliations:** ^1^Department of Bio Medical Engineering, Samrat Ashok Technological Institute, Vidisha, India; ^2^Department of Electronics and Instrumentation Engineering, Samrat Ashok Technological Institute, Vidisha, India; ^3^Department of Electronics and Communication Engineering, G. B. Pant Government Engineering College, Delhi, India

## Abstract

Ultrasound (US) image segmentation methods, focusing on techniques developed for fetal biometric parameters and nuchal translucency, are briefly reviewed. Ultrasound medical images can easily identify the fetus using segmentation techniques and calculate fetal parameters. It can timely find the fetal abnormality so that necessary action can be taken by the pregnant woman. Firstly, a detailed literature has been offered on fetal biometric parameters and nuchal translucency to highlight the investigation approaches with a degree of validation in diverse clinical domains. Then, a categorization of the bibliographic assessment of recent research effort in the segmentation field of ultrasound 2D fetal images has been presented. The fetal images of high-risk pregnant women have been taken into the routine and continuous monitoring of fetal parameters. These parameters are used for detection of fetal weight, fetal growth, gestational age, and any possible abnormality detection.

## 1. Introduction

There are various types of imaging modalities available such as ultrasound system, CT scan, MRI, NMR, and X-rays. There are various display modes, but the brightness mode ultrasound is the most normally applied investigative tool due to its noninvasive nature, cheaper cost, and small risk to the patient compared to other image modalities [1]. Generally, in radiology, injections such as radio-opaque dyes are needed, but in US, imaging external source is not required [2]. For diagnosis, the images of the organ are the most powerful technique for the obstetrician and gynecologist [3]. US image is molded when the satisfactory beam of sound waves is sent through the transducer in the human body. Received echo by the replication from internal organs creates appropriate ultrasound images. Moreover, due to properties of image formation, they could be influenced by the speckle, attenuation, missing boundaries, and artifacts, making the segmentation assignment more complicated [4].

The National Consensus for Medical Abortion in India report specified due to complications related to abortion each year an average of about 11 million abortions occur annually and around 20,000 deaths due to complications related to abortion [5]. Precise fetal parameter dimensions of US images are key issues for the pregnant woman's better health care. In obstetrics, fetal biometric parameters and thickness of nuchal translucency are essential parameters for the detection of fetal abnormality. The fetal biometric parameters include gestational sac (G.Sac), biparietal diameter (BPD), head circumference (HC), abdominal circumference (AC), and femur length (FL). These biometric parameters are used to measure the gestational age of the fetus and detect the growth patterns and abnormalities [1].

The nuchal translucency (NT) thickness of the fetus at 11–14 weeks of gestation was used to diagnose chromosomal abnormality [6]. NT thickness is the fluid accumulation in the nuchal region in the first trimester. Extensive research has verified that Down syndrome is a specific disorder triggered by the presence of an additional chromosome on chromosome 21. Generally, every human cell comprises 23 pairs of diverse chromosomes. Every chromosome transmits genes which are desirable for appropriate growth of human bodies. For the duration of conception, a specific receives 23 chromosomes each from the mother and the father. Children may receive the additional chromosome from any one of the parents. The latest study demonstrates that such fetal chromosomal anomalies can be sensed by measuring the NT thickness in the first trimester. The normal and abnormal growth is detected through measurements with the population-based growth chart. Manual measurements of fetal parameters are subjected to inter- and intraobserver variability [7]. Automatic methods for fetal parameter measurement reduce the inconsistency and create more accurate and reproducible measurements [8, 9]. Automated fetal monitoring improves the workflow efficiency; it helps to efficiently measure the fetal parameter. These accurate measured parameters will help the radiologist to diagnose the status of the fetus [10].

**Figure 1:**
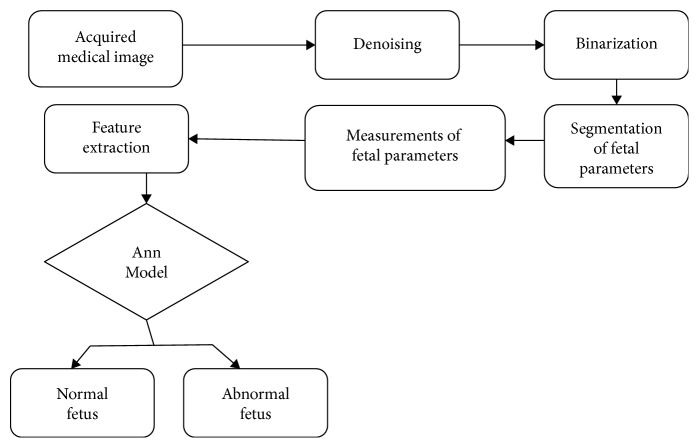
Process flow diagram for fetal growth detection.

An examination of medical images including image acquisition, enhancement, segmentation, compression, and storage of the measurement of anatomical and physiological parameters is presented [11]. The segmentation process of images gives qualitative and quantitative image analysis. The weak edges and wrong edges are inherent in the US images. It is more problematic to correctly segment the images. Many reviews [12–17] on image segmentation have been published in different journals, but none focused on the segmentation of 2D ultrasound fetal medical images.  Figure 1 shows the process flow diagram for fetal growth detection.

Three-dimensional medical ultrasonography was described in the early 1990s for fetal screening, but its spread was inadequate due to poor image class and slow acquisition protocols, unable to prevent fetal motion artifacts [18, 19]. These limitations are gradually vanishing with cutting-edge technologies, increasing the clinical interest in 3D ultrasound (3DUS). During the first trimester and early stage of the second trimester of gestation, the field of view of the ultrasound probes can integrate the whole gestational sac. Consequently, 3DUS-based volumetric studies of uterine structures have been published [20], as well as quantification of the whole fetus [21] or partial body portions (e.g., head and trunk) [22], providing useful information for clinical routine. These volumetric studies still rely on manual tracing, and automated segmentation methods are, therefore, desirable. Semiautomated methods were used in recent studies, especially with the software tool VOCAL, commercialized by General Electric and cited in several works [21–23]. It enables to reconstruct smooth organ surfaces from a set of 2D contours acquired on rotated views along a single axis [24]. This software remains limited to the extraction of single organs and is not yet capable of segmenting complex objects such as the whole fetus. Moreover, several manual interactions are often needed. The cost of 3DUS is also very high, so generally radiologists prefer 2DUS. In this paper, we focus only on 2DUS.

This paper is organized as follows.  Section 1 is the introduction of the automated techniques for the interpretation of fetal abnormalities; Section 2 describes the enhancement techniques of US images.  Section 3 summarizes the segmentation techniques of measuring the fetal parameters.  Section 4 presents the future trends of segmentation techniques. Finally, concluding remarks and suggestions for further development are outlined in Section 5.

## 2. Preprocessing of Ultrasound Images

Enhancements of US images are essential in manual assessment as well as computer-based analysis. US images are formed due to the pulse-echo system so the discrimination between normal and abnormal regions is complicated. The echoes received by the transducer are to be subject to the characteristic impedance of the medium:

(1) $$\begin{array}{c} I_{reflect}=I_{incident}{\left[\frac{Z_{1}-Z_{2}}{Z_{1}+Z_{2}}\right]}^{2}, \end{array}$$

where *Z*_1_ and *Z*_2_ are the characteristic impedance of the medium, *I*_reflect_  is the reflected ultrasound beam (echoes), and *I*_incident_ is the incident beam.

Procedures for contrast enhancement of ultrasound images are well known. The radiologist receives US images which contain arbitrary variations due to the statistics of echoes produced from the object. The detection of small and slight structure is difficult due to noises [25]. Speckle noises are formed from backscattered echoes randomly dispersed in the tissue [26, 27]. Because of the speckle presence, radiologists sometime fail to reach the conclusion [28]. The presence of speckle noise in the US images bounds its application in medical imaging. As a result, edge preservation [29] and enhancement [30, 31] are an essential operation in ultrasound image processing. The images are enhanced by applying various statistical filters [32], and the results are proven by measuring different parameters.

(2) $$\begin{array}{c} g\left(p,q\right)=f\left(p,q\right)-{\text{median}}_{\varrho }\left(p,q\right), \end{array}$$

where *g*(*p*, *q*) is the estimated local contrast. The indigenous contrast delivers high-frequency noise; *f*(*p*, *q*) is the image gray level and median_*ϱ*_(*p*, *q*) is the median gray level inside the region *ϱ*  of (*p*, *q*). Eq. (2) can be equated to a high-pass spatial filter. A Bayesian estimator-based discriminator for the improvement of images by extrication of image and noise was proposed [33]. It is a semiblind noise removal algorithm founded on a steerable wavelet pyramid.

## 3. Extraction of Fetal Parameters by Segmentation Techniques

In obstetrics [3], the fetal biometric parameters and nuchal translucency are the key parameters to indicate any possible abnormalities in the fetus. The normal growth of the fetal body indicates the changes in shape across gestation weeks of the fetus.

### 3.1. Fetal Biometric Parameters

The US system is noninvasive in nature, so continuous fetus monitoring is safe to use in the obstetric field. Assessment of the growth of the fetus and diagnosis of the fetal abnormality is easy using the segmentation process in image processing. Mostly, the image investigation is based on 2-dimensional B-mode US images. Among all biometric fetal parameters, head and abdomen segmentation is simple because of texture similarities and clear boundaries. The femur of the fetus can lack internal texture which makes the extraction more difficult. Abdomen and whole fetus segmentation is harder due to inconsistencies in the internal structures. The fetal biometric parameter measurement methods are used limitedly in clinical practice [9, 34–36].

#### 3.1.1. Probabilistic Boosting Tree (PBT)

PBT classifiers are represented by the nodes of a binary tree. Binary classification of data sets is automatically clustered by PBT [37]. Carneiro et al. [34] automatically detected the fetal parameters by the segmentation process applied on US images. The fetal parameters were also measured by ultrasound images based on the development of a constrained probabilistic boosting tree. In this work, automatic measurement of BPD, HC, AC, and FL of the fetus has been presented. They patented and developed a marketable system, called auto-OB [9]. This system used in clinical practice is the only system for measuring the fetal parameters.

#### 3.1.2. Fuzzy Logic

Fuzzy logic is an exceptional methodology applied across ultrasound images due to the fact that it does not require exact and enhanced images. In 1996, a semiautomatic fuzzy decision system developed for examination of the fetus has been presented. The system relies on the enhancement of the acquired images, which follows the decisional algorithms in the form of a sequence of If-Then rules. After acquiring the raw image and converting it into a desired format, various image processing algorithms are applied to analyze the images and measure the femur length, head circumference, and abdominal circumference [38]. Further, fetal biometric parameters are measured and analyzed by the maximum likelihood (ML) criterion algorithm, as proposed by Jardim and Figuiredo [39]. Manual extraction of contour in medical images requires expert knowledge and higher processing time. Fetal biometric parameters are measured for the detection of gestational age by a class-separable shape-sensitive approach [40]. In this approach, too many cost functions are assumed, which shows both its limitations and complications. The cost and objective function is the mathematical expression for the shape-sensitive derivative approach. The cost function at a different pixel level of the image is given by [41]

(3) $$\begin{array}{c} P_{ec}=\frac{\varepsilon \left(p\right)+4\lambda C_{f}\left(p,d\right)}{1+4\lambda }, \end{array}$$

where *C*_*f*_ is the cost function and *λ* is the weighting factor. The value of *λ* depends on the intensity of images and the number of classes.

#### 3.1.3. Thresholding-Based Morphological Operator

**Figure 2:**
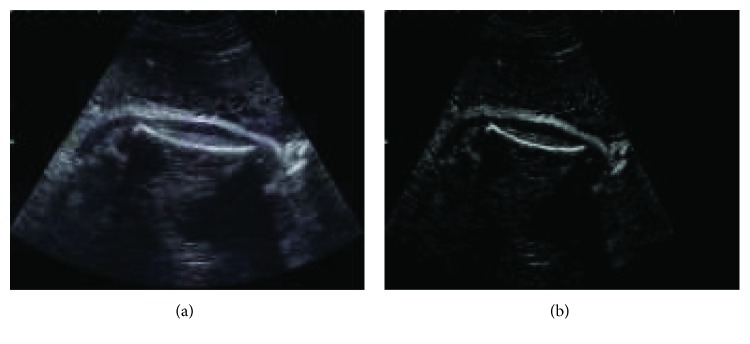
(a) Original 24-week femur region image. (b) Femur region superimposed onto the original image.

The femur of the fetus is segmented through the morphological operator, then the length of the femur is measured. Thomas et al. [42] proposed the morphological feature-based algorithm to detect the contour of the femur in US images and automatic length measurement of the femur bone in the fetus. Further, in 2009 [43], gestational age of the fetus was measured using a femur length. Rawat et al. [44] estimate the fetal weight using the femur length as shown in Figure 2; the weight of the fetus is compared with a gold standard, and then the abnormality in the fetus is predicted.

The length of the femur is also measured by applying the morphological operator; by this approach, automatically the FL of the fetus is measured. They projected two methods to extract the femur bone of the fetus: one is based on the entropy approach and another on edge detection. The entropy-based method is the main approach, and when the first one is failed, then the second method was only used.

#### 3.1.4. Gradient Vector Flow (GVF) Methodology

The GVF snake is a segmentation approach [45] which has been effectively used in the segmentation of medical images. The contour of a snake [46] does not converge to the object boundary. In the image domain, the contour is initialized by the operator and then the boundary is formed in an object. According to the differential equation of GVF, the modified form of the elastic contour is defined as an external force. The vector field of the two-dimensional function is *r*(*X*, *Y*) = (*p*(*X*, *Y*) + *q*(*X*, *Y*)) which minimizes the following objective function:

(4) $$\begin{array}{c} E=\iint \mu \left(p_{x}^{2}+p_{y}^{2}+q_{x}^{2}+q_{y}^{2}\right)+{\left|\nabla f\right|}^{2}+{\left|r-f\right|}^{2}dxdy, \end{array}$$

where *E* is the energy function, *p*_*x*_, *p*_*y*_, *q*_*x*_, *q*_*y*_ are the field derivatives, *μ* is the regularization parameter, and *∇f* is the gradient of the edge map. Chalana et al. [47–49] report an active contour model for segmentation of the fetal head and abdomen in the US images. In the physical correction in the image, it can get trapped in the local minima. Also, due to the texture inside the fetal head, the algorithm does not make the model which means that the appearance information is not used to change the accuracy. Jardim and Figueiredo [50] report the parametric deformable shape methodology for the segmentation of fetal parameters. A weakness of this method is that the optimal solution of the problem does not assure the observation of the authors. Another drawback is that the Rayleigh distribution-based model cannot take into account the spatial structure of textural patterns. The wavelet-based techniques [51] and iterative Hough transforms [52] are also useful in extracting the object or segmenting the fetal images.

In 2008, the abdominal circumference is measured by the fuzzy and gradient vector flow (GVF) methods. In the GVF method, an active contour is formed and the GVF field behaves as an external force. After applying the above method, the fetal weight is estimated using the abdominal circumference; the comparison between the accuracy of the automatic and manual measurements were presented [53]. Further, a GVF snake is reported by Nithya and Madheswaran [54] to form or extract the contour of the abdominal circumference. The value of the abdominal circumference is used to detect the intrauterine growth retarded (IUGR) fetus. The IUGR fetus is at higher risk for slow development, abdomen problem, cardiac disease, and other problems in adult life. Yang et al. [55] detected the fetal head region using the Hough transform-based classifier. In this work, a quadratic polynomial model $\;\tilde{HC}$ used to assess the HC using least square fitting methods is defined as

(5) $$\begin{array}{c} \tilde{HC}=p_{1}z^{2}+p_{2}z+p_{3}, \end{array}$$

where *z* indicates the gestational age, and *p*_1_, *p*_2_, and *p*_3_ are the coefficients. Further, the manual and automatic results are compared and it is concluded that the difference between them is not considerable.

**Figure 3:**
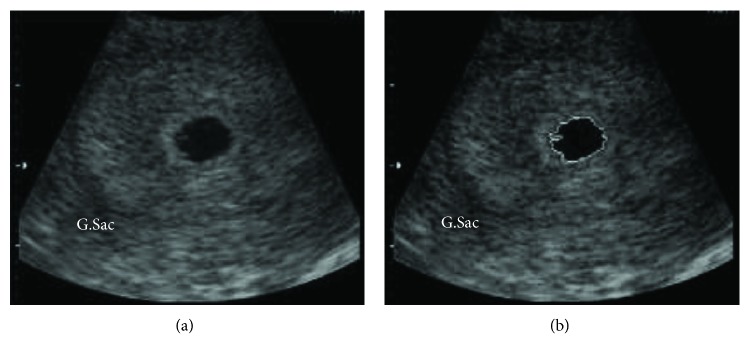
(a) Original 6-week and 4-day gestational sac image. (b) G.Sac contour formed using gradient vector flow (GVF) snake.

In 2014, authors applied various segmentation method for assesssment of fetal femur, fetal head and abdomen. They evaluate the results on the basis of region-based metrics which is verified by various experts [13]. Ponomarev et al. [14] applied resulting binary images with combined numerous thresholds, edge detection, and shape-based recognition. The gestational sac diameter has been used as the first fetal parameter for confirmation of pregnancy. Chakkarwar et al. [15] worked for finding the diameter of G.Sac. In this work, two steps are followed: in the first step, the global thresholding technique was used [16], then in the second step the diameter of G.Sac was measured. Rawat et al. [17] proposed the GVF methodology for finding the G.Sac contour and measuring the diameter of G.Sac. Then, the G. Sac region of the fetus is automatically segmented from the whole image and the G.Sac diameter has been measured as shown in Figure 3.

#### 3.1.5. Graph-Based Approach

The graph-based method is proposed to extract the head of the fetus by a semisupervised patch-based approach [56]. Many segmentation problems are solved by a fast minimization format and a nonstop min-cut divider [57] in the graph. In this method, an initial label has to be defined on every image since the method is semisupervised. Fetal BPD and OFD are measured by a graph-based approach called the circular shortest path (CSP) which is a fast automatic approach [58]. Authors have done the qualitative and quantitative analysis of the segmented results which have been verified by experts.

### 3.2. Nuchal Translucency

Nuchal translucency (NT) of the fetus is also an important parameter for the diagnosis and assessment of fetuses. The fluid accumulation in the nuchal region at the first trimester of the fetus is the NT thickness [59]. Down syndrome in the fetus is detected by NT thickness, so large NT thickness indicates an abnormal condition. Down syndrome fetus and trisomy of 13, 18, and 21 at 10–14 weeks of gestational age have 3 mm NT thickness [60]. The bigger NT thickness indicates the structural defects and genetic syndromes even in the normal karyotype fetus [61]. NT thickness in the 10–14 weeks of gestation has been evidenced to be one of the most perceptive parameters [62].

**Figure 4:**
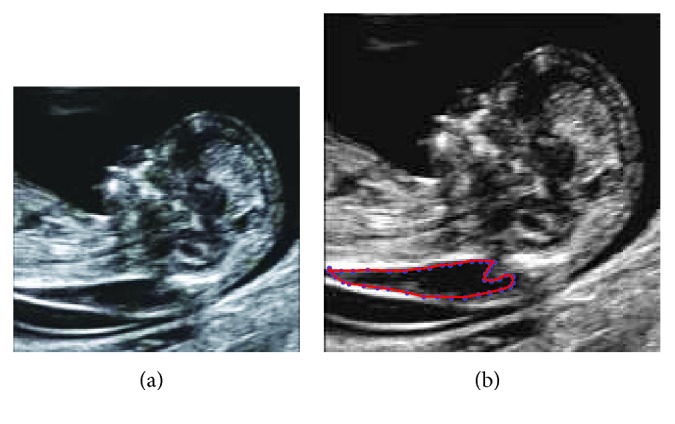
(a) Original 12-week fetus image; (b) abnormal NT thickness.

An automatic scheme is proposed by Deng et al. [63] to estimate the fetal thickness of NT, using a filtering technique. In this technique, the initial contour is first created and extracts a preliminary contour by the GVF methodology. Then, for finding the final NT contour and computing the edge map, dynamic programming is used. Finally, NT thickness and the NT area of the fetus are calculated as shown in Figure 4.

Nirmala et al. [64] measure the thickness of NT to recognize chromosomal abnormalities in the first trimester fetus in three steps. In the first step, the preprocessing techniques are applied for filtering the images; in the second step, mean shift analysis [65] has been done for segmenting the NT region. Next, Canny operators for edge detection have been applied and by Blob analysis the exact thickness of the NT has been predicted. All segmentation techniques which are used for segmenting the fetal parameters are described in Table 1, and comparison of all methods are described in Table 2.

## 4. Future Trends Based on the Supervised Learning Method

Previously, an assortment of the segmentation algorithm such as threading [66] and edge detection [67–72] techniques have been applied on ultrasound images, for extracting the fetal parameters. The segmented region has simple and spatially accurate boundaries. This accomplishes major difficulties, since ultrasound medical images have a small hole and boundaries are also irregular. The following may be the future segmentation trends, for achieving the accurate detection task from fetal ultrasound images.

### 4.1. Neural Network Based on the Hybrid Approach

The neural network-based approach has been generally used in the medical field for diagnosis [73]. In the diagnosis, raw data obtained from patients are evaluated and then various artificial intelligence techniques are applied for classification or detection. Chuang et al. [74] proposed the artificial neural network (ANN) model for assessment of fetal weight and concluded that the errors are less between the calculated fetal weight and the actual fetal weight. The weight of the fetus is the indication of anomaly finding in the fetus. The accurate weight of fetus measurement is a desirable task, although previously the ANN model is used for fetal analysis, which belongs to the macrosomia group [75]. Further, the ANN model [76] is designed for diagnosis of IUGR disease in the fetus. Khashman and Curtis [77, 78] proposed the neural network model for edge detection of the fetal head and abdomen automatically. Previously, the backpropagation algorithm is applied for detection of fetal anomaly based on the head and abdominal circumference [79]. In 2011, Anjit et al. [80] proposed the ANN model for extraction of the fetal parameter of the nasal bone region of US images. Nasal region parameters are extracted in the spatial domain and converted into the spatial domain by using discrete cosine transform and wavelet transform. The training of these networks consists of mapping between an input data and a set of output data. This mapping is trained by adjusting the weights by learning the algorithm followed by the generalized delta rule [81]. In the ANN model, weights are adjusted on the training set then their value is stable and the unknown input vectors are classified. According to the generalized delta rule, the error term minimization is defined as

(6) $$\begin{array}{c} E_{K}=0.5*\sum \left(t_{k}-y_{k}\right)2. \end{array}$$

In this equation, the index *K* represents the input vector, and *t*_*k*_ is the target vector and *y*_*k*_ are the actual output vectors.

(7) $$\begin{array}{c} \Delta w_{jk}=\eta \partial_{k}z_{j} \end{array}$$

where  *η* is the rate of learning, *∂*_*K*_ is the local gradient, and Δ*w*_*jk*_ is the change in weight from node *j* to *k*.

In 2014, the authors presented a new hybrid approach for detection of the IUGR fetus, using the variational level set method. Level set methods [82] are applied across fetus images for measuring the BPD and head region. The BPD and head circumference values are the test data for classification problem in the neural model. An enhanced MLP network is presented for the detection and classification of the IUGR fetus [83]. The accuracy of the IUGR fetus is calculated by measuring the statistical parameter. A multilayer perceptron network with the hybrid approach is widely used in medical image segmentation [84–87].

### 4.2. Support Vector Machine (SVM) Approach

SVM is a classification technique for a two-group categorization problem proposed by Cortes and Vapnik [88]. The SVM model separates the positive classes (+1) and the negative classes (−1) by an optimal hyper plane. The separation between the two classes is maximized by finding the linear optimal hyper plane [89].

The SVM model in an object has M training data points  {(*p*_1_, *q*_1_), (*p*_2_, *q*_2_),…, (*p*_*M*_, *q*_*M*_)}, where *p*_*M*_∈ real integer and *q*_*M*_∈{+1, −1}. In the SVM algorithm, the hyper plane is indicated by (*w*, *b*) where *w* is the weight vector and *b* is the bias; *x* is the object classifier. In the SVM model, the data is not linearly separable, then nonlinear data points are changed to the higher-dimensional space; the data points then become linearly separable.

In 2014, Qasem et al. [90] proposed the radial basis function (RBF) kernel for breast cancer mass identification in the images. For the diagnosis of breast cancer, first of all apply segmentation algorithm across breast US images. Then, the breast images are evaluated on the basis of comparison with the ground images. Each pixel in the resulting image is compared with the equivalent pixel in the ground images firstly. Then, the confusion matrix is calculated from the resulting image with and without the use of the rejection model. Further, Hassanien and Kim [91] introduce a fusion approach that associates the fuzzy logic, SVM model, pulse coupled neural networks, and wavelet-based algorithm. In the MRI images, the SVM classifier gives the result in two categories: the first is cancerous and the second is noncancerous. Comparing with other classifier SVMs gives a more accurate result.

## 5. Conclusions

In this paper, a segmentation evaluation of current trends for fetal parameters is briefly reviewed. The fetal parameters can give the prediction of fetal abnormality, so accurate measurement of these parameters is of prime concern. After discussions and various simulation results were obtained, we find that the shape of fetal parameters is different, so the GVF contour method is excellent for elliptical shape parameters (AC, HC, BPD, and NT region) and morphology-based techniques are good for measuring the femur length of the fetus. A graphical approach is found better for the femur and head contour measurement of the fetus. After feature extraction, the classification techniques (neural network and support vector machine) are applied in predicting the abnormalities of the fetus. The high-risk pregnancies can be detected easily by the precise monitoring of the fetus with time and is more accurate using automated segmentation techniques. Computer-based techniques are accurate, and the speed of the algorithm is also very fast. But in the case of multiple or twin pregnancy, the parameters are not detected easily and iteration time and computational time are larger in the active contour method.

Current trends are based on an advance contour algorithm for segmentation, and a neural network-based hybrid approach and support vector machine classifier may be applied for fetus abnormality prediction. In future research, the diagnosis of medical images by the segmentation process and artificial neural model will help in improving the accuracy, precision, and computational speed. The computational-based approach also reduces the manual interaction. Further research is based on early and accurate detection of fetus status at a cheaper cost. The health care system and equipment are enhanced by the advance techniques for assisting the radiologist in making decisions effectively.

## Figures and Tables

**Table 1:** Overview of ultrasound image segmentation techniques. A listing of popular feature extraction and classification methods for fetal US.

Author	Year	Methodology used	Fetal parameter	References
Thomas et al.	1991	Thresholding-based morphological operator	FL	[42]
Smith and Arabshahi	1996	Fuzzy decision system	HC, AC, FL	[38]
Chalana et al.	1996	Active contour model	BPD, HC	[47–49]
Gurgen et al.	1996	Neural Network	HC/AC ratio and IUGR fetus	[76]
Zayed et al.	2001	Wavelet transform	Biometric parameters	[51]
Jardim and Figuiredo	2003	Maximum likelihood criteria	Biometric parameters	[39]
Jardim and Figueiredo	2005	Deformable shape model	BPD, FL	[50]
Zoppi et al.	2005	Gradient vector field snake	NT parameters	[59]
Carneiro et al.	2008	Constrained probabilistic boosting tree	Biometric parameters	[9, 34, 35, 37]
Jinhua et al.	2008	Gradient vector field snake	AC	[53]
Shan and Madheswaran	2009	Class-separable sensitive approach	Biometric parameters	[40]
Nithya and Madheswaran	2009	Gradient vector field snake	AC and IUGR fetus	[54]
Shrimali et al.	2009	Thresholding-based morphological operator	FL	[43]
Nirmala and Palanisamy	2009	Edge detection algorithm	NT thickness	[64]
Rawat et al.	2011	Thresholding-based morphological operator	FL and fetal weight	[44]
Anjit et al.	2011	BPNN-based neural network	Nasal bone of fetus	[80]
Wang et al.	2012	Entropy and edge detection-based technique	FL	[92]
Ciurte et al.	2012	Graph-based approaches	HC, AC	[56, 57]
Sun	2012	Graph-based approaches	HC	[58]
Choong et al.	2012	Variational level set-based neural network	Fetal size	[83]
Rawat et al.	2013	Gradient vector field snake	G.Sac	[17]
Rueda et al.	2013	Difference of Gaussian revolved elliptical path, boundary fragment model, multilevel thresholding	HC, AC, FL	[13]
Yang et al.	2013	Neural network based approach	HC	[55]
Gadagkar and Shreedhara	2014	Variational level set-based neural network	Fetal size and HC, AC, and IUGR fetus	[82]

**Table 2:** Comparative analysis of important fetal image segmentation techniques.

Segmentation techniques	Advantage	Limitation	References
Constrained probabilistic boosting tree	The results are based on the tree structure, so segmented biometric parameters are measured accurately.	The process of multistage decision and the data input is in binary form.	[9, 34, 35]
Fuzzy decision system	The detection is based on fuzzy boundary, and all parameters are boundary sensitive.	The fuzzy system is based on a series of If-Then rules, making the system complicated.	[38]
Class-separable sensitive approach	The fetal biometric parameter shape is of different types so the class-separable approach is good.	US image is having some noise, and it is very much sensitive to noise.	[39, 40]
Thresholding-based morphological operator	Advantage of thresholding lies in its simplicity, which involves minimal implementation and computational requirements.	It is sensitive to noise, and it cannot be an effective segmentation technique for US medical images.	[14, 66, 67]
Edge detection algorithm	The amount of data to be processed is reduced, and the analysis of images is simple. Besides, at the same time it preserves useful information about object boundaries.	The masks used by different operators act as a high-pass filter, which tend to amplify the noise.	[67, 68, 77, 78]
Active contour model	It can generate the closed parametric curve directly from the images by calculating the external force. It also includes the robustness against the noise (internal force).	The initial contour is placed manually, so the method is sensitive. Problems are associated with initialization of contour and convergence to their boundary concavities.	[52–54, 14, 63]
Wavelet transform	This approach is based on the texture of the object so the results are accurate.	The fetal parameters is of various sizes so sometimes the discrimination is emblematic.	[51]
Graph-based approaches	This approach is good because the whole image is considered and the evaluation of parameters is closer to the expert results.	In this approach, few clicks are placed manually for continuous min-cut partition of the graph.	[13, 56–58]
Neural network	The NN can be applied to any classification/recognition problem by modifying only the training set. So easily the network can be trained.	There are various types of classifier used in NN, so the selection of a proper algorithm and classifier gives good results.	[77–79, 83, 84]
Level set	All level sets yield a nice representation of regions, without the need of a complex data structure.	A level set function is restricted to the separation of two regions. As soon as two regions are considered, the level set idea loses part of its attractiveness. Results vary due to initial contour placement.	[81, 82]
